# Differential Diagnostics of Pain in the Course of Trigeminal Neuralgia and Temporomandibular Joint Dysfunction

**DOI:** 10.1155/2014/563786

**Published:** 2014-06-04

**Authors:** M. Pihut, M. Szuta, E. Ferendiuk, D. Zeńczak-Więckiewicz

**Affiliations:** ^1^Department of Dental Prosthetics, Consulting Room of Temporomandibular Joint Dysfunctions, Medical College, Jagiellonian University, 4 Montelupich Street, 31-155 Krakow, Poland; ^2^Cranio-Maxillofacial Surgery, Medical College, Jagiellonian University, 1 Zlotej Jesieni Street, 31-826 Krakow, Poland; ^3^Department of Dental Surgery, Wroclaw Medical University, 26 Krakowska Street, 50-425 Wroclaw, Poland

## Abstract

Chronic oral and facial pain syndromes are an indication for intervention of physicians of numerous medical specialties, while the complex nature of these complaints warrants interdisciplinary diagnostic and therapeutic approach. Oftentimes, lack of proper differentiation of pain associated with pathological changes of the surrounding tissues, neurogenic pain, vascular pain, or radiating pain from idiopathic facial pain leads to improper treatment. *The objective of the paper* is to provide detailed characterization of pain developing in the natural history of trigeminal neuralgia and temporomandibular joint dysfunction, with particular focus on similarities accounting for the difficulties in diagnosis and treatment as well as on differences between both types of pain. It might seem that trigeminal neuralgia can be easily differentiated from temporomandibular joint dysfunction due to the acute, piercing, and stabbing nature of neuralgic pain occurring at a single facial location to spread along the course of the nerve on one side, sometimes a dozen or so times a day, without forewarning periods. Both forms differ significantly in the character and intensity of pain. The exact analysis of the nature, intensity, and duration of pain may be crucial for the differential diagnostics of the disorders of our interest.

## 1. Introduction

According to the definition provided by the International Association for the Study of Pain, pain is a subjectively unpleasant and negative sensory and emotional experience occurring following activation of nociceptive stimuli that damage the tissue. The character of pain depends on its location, type of dysfunction of the particular region, and stage of the disease. It is also an observation made during mental interpretation of associated phenomena. Although pain is associated with unpleasant sensations, it also plays a positive forewarning and protective role. Pain is an extremely complex neurophysiological process [1–12]. It appears as the result of a damaging stimulus and the effect of tissue hormones (serotonin, bradykinin, histamine, leukotriene, and accumulation of hydrogen ions) on nociceptors, that is, receptors specialized in the reception of pain and discomfort sensations. Nociceptor excitability depends on physical stimuli, physiochemical milieu, and the quantities of endogenous pain substances being secreted [2–6]. Factors such as microcirculation, dysregulation of the sympathetic nervous system, and excessive muscle tone affect the activity of pain receptors. The process of the development of pain sensation is known as nociception and consists of four stages: transduction, transmission, modulation, and perception. Nociceptive stimuli are transmitted by the neuronal route of the posterior spinal horn and by the spinothalamic routes to cortical centers where perception of pain sensations occurs [1, 2].

The objective of the paper is to provide detailed characterization of pain developing in the natural history of trigeminal neuralgia and temporomandibular joint dysfunction, with particular focus on similarities accounting for the difficulties in diagnosis and treatment as well as on differences between both types of pain.

## 2. Craniofacial Pain

Chronic oral and facial pain syndromes are an indication for intervention of physicians of numerous medical specialties, while the complex nature of these complaints warrants interdisciplinary diagnostic and therapeutic approach. The incidence of oral and facial pains is estimated at 10% in adults and 50% in elderly patients. Oftentimes, lack of proper differentiation of pain associated with pathological changes of the surrounding tissues, neurogenic pain, vascular pain, or radiating pain from idiopathic facial pain leads to improper treatment. Diseases that involve significant pain, when inappropriately treated, lead to the reduction in the quality of life and to the development of depressive disorders [2–14].

Of the important data provided in medical interviews, particular attention should be drawn to the time of the onset of pain, pain location and characteristic, intensity, frequency and factors that enhance or reduce the intensity of pain. Also important is the radiation of pain to the surrounding organs of anatomical structures [1–15].

Pain within the craniofacial area is one of the most important reasons why patients present at the dentist's office [2–11]. Odontitis, periodontopathies, alveolar osteitis, nerve injuries, atypical facial pains, neoplastic lesions, elongated styloid process syndrome (Eagle's) syndrome, and reflex sympathetic dystrophy of the face should be taken into account. Common causes of pain include trigeminal neuralgia (most commonly of the third branch of the trigeminal nerve, i.e., the mandibular nerve) and temporomandibular joint dysfunction. In the differential diagnostics of facial pains, disease duration of several months or several years required unified diagnostic criteria with consideration of nontypical cases. In the therapy, we use various methods of treatment such as medication, surgery, dental prosthetic, physiotherapy, and psychological support [2–36].

## 3. Trigeminal Neuralgia

Neuralgia is a symptom of nerve dysfunction present within the brain stem or within the nerve segment running to the trigeminal ganglion located within the base of the middle cranial fossa. The disorder is most common in patients over 60 years of age and more common in women. Main etiological factors responsible for neuralgia include vasculoneural conflict consisting in compression of the nerve by blood vessels at the site of neural connection to the brain stem, within the region of the superior cerebellar artery, the basilar artery, the vertebral artery, and the petrosal vein. In addition, neuralgia may be a result of head injuries or inflammation of nerve within the myelin sheath. The disorder may also be associated with other diseases such as multiple sclerosis (formation of demyelinating plaque in the brain) or tumors that compress the nerve and disturb its function [4, 8–17, 23]. According to the most recent theory of magnetic bioresonance, trigeminal nerve starts oscillating at amplitudes that exceed its natural frequency. The human organs differ in their natural frequencies (8–12 Hz for the head). The nerve is immersed in the cerebrospinal fluid that transmits the vibrations of the surrounding structures [5, 9]. The increase in the amplitude of vibrations damages the permeability of ion channels, leading to nerve injury. The disorder is characterized by recurrent, paroxysmal attacks of sudden, intense, and piercing pain within the region supplied by the trigeminal nerve, comparable to an electric shock [5–7, 9–19, 30].

It might seem that trigeminal neuralgia can be easily differentiated from temporomandibular joint dysfunction due to the acute, piercing, and stabbing nature of neuralgic pain. It should be mentioned that neuralgia may be of either spontaneous (primary) or symptomatic (secondary) form. Both forms differ significantly in the character and intensity of pain. Unexplained etiology of spontaneous neuralgias of the second and third branch of the trigeminal nerve is a cause of significant therapeutic problems [1–8, 10–15]. Both branches may also be affected at the same time. The incidence is 1 in 15,000 cases. The acute, paroxysmal, and piercing pain occurs at a single facial location to spread along the course of the nerve on one side, sometimes a dozen or so times a day, without forewarning periods. Pain episodes may last several seconds to several minutes. As the disease progresses, the number of episodes increases and the remission periods become shorter. The pain is intensified during facial muscle and mandibular movements. Primary neuralgia is often accompanied by facial muscle contractures (*tic douloureux*), increased salivation, lachrymation, running nose, and skin redness. Stimuli that most commonly cause the pain are trivial and everyday causes such as wind gusts, sudden changes in air temperature, bright light, sharp sounds, or delicate touch (e.g., shaving in males). A typical feature of these disorders is the unilateral occurrence of pain and lack of complaints during the night's sleep [3, 5, 7, 9–13, 15].

In contrast to the spontaneous neuralgia, its symptomatic (secondary) form is associated with pain that increases gradually, has different nature, and persists with no interruptions. The pain becomes intensified in heat. It may be a result of numerous local or generalized causes (odontitis, cysts, sharp socket edges, tumors within the mouth, and the maxillo-ethmoidal massif, disorders of the maxillary sinuses or the middle ear). Neuralgia of this type may be a symptom of numerous diseases within the region of the posterior cranial fossa, such as basal tumors or cerebellopontine angle tumors. This type of neuralgia may also be observed in alcohol, mercury, or nicotine intoxication [8–13, 15, 18, 30].

Symptomatic neuralgia should be differentiated from causalgia which may be due to the traumatic injury of nerve, particularly maxillary nerve, or mandibular nerve within the facial region. Causalgia may develop as a result of contusion, fracture, or surgical intervention in this region. As shown by the characteristic of pain, primary neuralgia is substantially different from the secondary form. Therefore, the pain associated with the temporomandibular joint dysfunction may have a characteristic that is similar to the secondary neuralgia and may pose significant difficulties in differential diagnostics [4, 5, 8–10, 13, 14].

## 4. Temporomandibular Joint Dysfunction

According to WHO report, temporomandibular joint dysfunction is the third stomatological disorder to be considered a populational disease, after dental caries and periodontal diseases. Temporomandibular joint dysfunction consists in a spectrum of changes disturbing the morphological and physiological balance within the musculoskeletal system. The nature of these changes is determined by psychoemotional, environmental, and genetic factors. The changes include abnormalities in the relationship between opposing teeth, and the function of the muscles of the frontal and medial part of the skull and neck, working in a symmetrical manner in physiological conditions of the temporomandibular joints. Increasing stress levels lead to intensification of adverse motion habits within the stomatognathic system and the rapid increase in the number of patients observed in recent years is associated with the drop in the age of patients with dysfunctions manifested with pain symptoms [14, 15, 17, 18].

Functional disorders of the masticatory organ are pathologies of diverse etiology [2]. The incidence of the painful form of the disorders is estimated at about 30% of all cases. Most commonly, pain is located within the temporomandibular joint region or, less commonly, the masticatory muscles (myalgia). It may range from slight tenderness to a very strong discomfort. The pain is either acute and stabbing or chronic, diffuse and radiating into the neighboring structures, that is, eyes, ears, temples, and occiput. It is not associated with inflammation; the intensity of muscle pain is closely related to excessive functional activity of particular muscle groups during occlusal parafunctions. Overburdened muscles are characterized by hypoxia and ischemia and thus with the release of allogeneic substances that determine the resultant pain, such as bradykinin or prostaglandins. Muscle tenderness is a source of deep pain and may lead to protective contractures [2–5, 18, 19].

The articular pain is a result of damage to articular surfaces, degeneration, injuries of articular capsule, or damage of retrodiscal tissue. Arthralgia may develop only as a result of impulsation originating from nociceptors located within the soft tissue. Lack of coordination of mandibular head and disc is manifested by acoustic symptoms within the temporomandibular joints, such as popping and cracking sounds upon mandibular movements. At advanced stages of disc translocation and blockade, acoustic symptoms disappear to be replaced by chronic pain and significant restriction of jaw opening range, complicated by tilting the mandible towards the affected side upon abduction. Temporomandibular pains may also be the result of prolonged overload of articular structures of high intensity, exceeding the adaptational capabilities of the collagen fibers within the posterior disc ligament, which are commonly subject to fragmentation. The nature of the pain observed in temporomandibular joint dysfunction is similar to that in the symptomatic form of neuralgia and significantly different from that in the spontaneous neuralgia [2, 15–28].

## 5. Common Features of Both Types of Pain

Pains sensations experienced in trigeminal neuralgia and temporomandibular joint dysfunction have both common features and significant differences (Table 1). Common features include pain being radiated into the neighboring regions, and even to distant structures, possibility of pain being located on one side of the face, increase in pain as a result of increased activity of facial or masticatory muscles and the possibility of otolaryngological symptoms (earache and hearing impairment) [16]. In addition, the diffuse pain that lasts for many months in both cases may lead to significant reduction in patients' quality of life and to the development of depressive disorders. Compared to primary neuralgia, pain experienced in secondary neuralgia is much more similar to that observed in functional disorders [2, 15, 17–20].

## 6. Differences in the Characteristics of Pain

Features differentiating both nosocomial entities include unilateral location of pain in neuralgia (97%) and bilateral pain in myalgia. Also the nature of pain is different, being acute and stabbing in neuralgia and continuous, dull in temporomandibular joint dysfunction. Night's rest is a period of remission of the neuralgic pain, while temporomandibular joint pain may still be present in this period in case of functional disorders [13–16]. The duration of pain is also different: the neuralgic pain is very short (lasting several seconds to several minutes), with long periods of remission during the day, while the dysfunctional pain is long-lasting (several hours) with short intermissions. Pain episodes in spontaneous neuralgia are induced by triggering stimuli which are usually the same in particular patient. Patients tend to avoid these stimuli and are afraid of them. If neuralgia episodes are induced by chewing, patients fast deliberately, which leads to secondary and multiplanar somatic disorders. Neuralgic pains are often accompanied by facial muscle convulsions (tics), skin redness, lachrymation, which are not observed in temporomandibular joint dysfunction. In neuralgia, pain is intensified by heat, while being alleviated in myalgia [2–5, 9–35].

Thanks to the development of neurophysiological examinations, magnetic resonance imaging, cerebral angiography, and clinical symptoms, trigeminal neuralgia may be correctly diagnosed while excluding other causes for paroxysmal facial pains. The exact analysis of the nature of pain may be crucial for the differential diagnostics of the disorders of our interest [3, 10, 22, 36–48].

**Figure 1:**
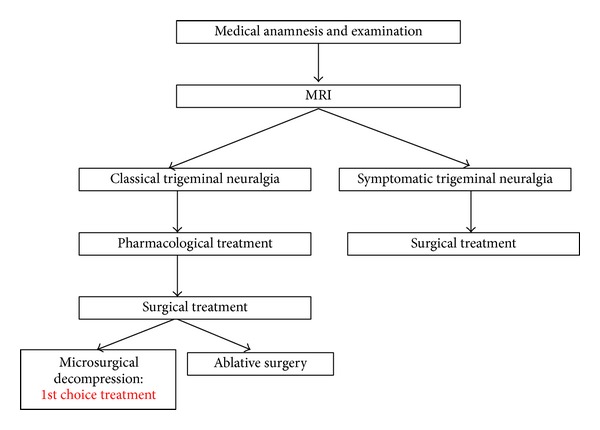
Diagnostic and therapeutic management of patients suffering from trigeminal neuralgia.

Despite similarities of pain symptoms in both types of pathologies, therapeutic management is different in both cases. In trigeminal neuralgia, treatment involves conservative and more or less invasive methods. Usually, the treatment is commenced carbamazepine (100 to 1000 mg/day) pharmacotherapy, in some cases combined with anticonvulsants (phenytoin, clonazepam). One should also keep in mind the ability to achieve long-term remission of neuralgic pains by means of neural blockades of the terminal trigeminal nerve branches using lidocaine or bupivacaine solutions (Figure 1). The next step consists in surgical treatment; however, the use of surgical methods, even in the form of minor procedures such as neurotomy or exeresis, requires that the patient should be qualified for the surgery by both internal medicine specialist and anesthesiologist which constitutes a major problem, considering the commonly elderly age of patients combined with significant burden of concomitant diseases [10, 36–39, 42].

The latest method is stereotactic surgery/gamma knife, making use of electromagnetic gamma radiation from cobalt ^60^Co isotope sources, corpuscular radiation of protons of heavy carbon ions, or electromagnetic X radiation generated by linear accelerators. The method was developed by professor Lars Leksel and has been known since 1967. The method consists in precise delivery of radiation from 192 collimator sources into intracranial pathological lesions using stereotactic techniques. The first stage of the procedure is the placement of stereotactic frame on patient's head using special screws in local anesthesia. The patient is placed within a gamma knife apparatus, where the irradiation spot location and dose magnitude is determined on the basis of magnetic resonance scan results and the location of the lesion. The usual dose is 90 Gy and is absolutely safe for tissues permeated by radiation. Irradiation lasts about 30 minutes. Factors taken into consideration when qualifying patients for the treatment include the type, size, and location of lesions. Radiosurgery techniques have witnessed an enormous technological progress. At the same time, a model of multidisciplinary teams of oncologists, radiotherapists, neurosurgeons, and other medical specialties has been developed for the radiosurgery purposes. The efficacy of stereotactic radiosurgery is estimated at 80–90%. Due to the minimum invasiveness of the procedure, it is the method of choice in elderly patients or patients with high concomitant disease load [42, 49].

Due to the poor availability of this treatment in Poland, microvascular decompression (MVD) technique is still in use, consisting of craniotomy followed by elimination of the vasculoneural conflict by means of separating the problematic artery or vain from the nerve using autogenic (muscle fragment) or alloplastic (teflon, goretex) material [5, 8, 10, 14, 32].

The natural history of temporomandibular joint dysfunction most commonly involves changes in the biomechanics of temporomandibular joints connected with the dysfunction of masticatory muscles. The treatment of functional disorders usually involves occlusal splints, characterized by multiple effects. These include elimination or reduction of pain by reducing the load on temporomandibular joints and retrodiscal structures as well as reduction of excessive activity and restoration of symmetry in the tone of masticatory muscles. This is known as reversible occlusal treatment. Prosthetic methods for the treatment of dysfunction include procedures to correct the occlusion system (selective contouring, reconstruction of correct occlusal conditions with fixed or mobile prostheses) [1–3, 5, 8, 18, 25–29, 41, 44, 47, 48].

Physiotherapy plays an important role in the treatment of patients with temporomandibular joint dysfunctions when used as an element of supportive therapy applied simultaneously to the primary treatment. The goal of physiotherapeutic procedures is to eliminate or reduce pain, reduce the excessive tone of overloaded muscles of the head, neck, and shoulder girdle, activate muscles of reduced muscle tone, and mobilize joints of limited mobility. The techniques used in physical therapy affect motor coordination of the musculoskeletal system and have beneficial effect on the increased vascular flow in the treated area. Physiotherapeutic methods used in stomatology include kinesitherapy, laser therapy, manual therapy, massage, light therapy, electrotherapy, and magnetoledtherapy [2, 15, 19, 21, 23, 33, 38].

A new pharmacological approach consists of the use of botulinum toxin type A in the treatment of excessive muscle tone which constitutes a serious problem in the natural history of the temporomandibular joint dysfunction [26, 28, 29]. The mechanism of action of the drug involves suppression of the release of acetylcholine (parasympathetic neurotransmitter) into the synaptic cleft and blockade of the transmission of impulses leading to muscle contracture. Intramuscular injections of the toxin are administered in the region of the largest cross section of the masseter muscle. The duration of the relaxant effect of the drug depends on the dose strength; most usually, it is 6 months [26]. There are several other medicines like Propolis or topical application of Ketoprofen in the area of temporomandibular joints [22, 23, 31].

## 7. Summary

Chronic oral and facial pain syndromes are an indication for intervention of physicians of numerous medical specialties, while the complex nature of these complaints warrants interdisciplinary diagnostic and therapeutic approach. The precise analysis of characteristic features of pain, its intensity, and duration may be crucial for the differential diagnostics of trigeminal neuralgia and functional disorders of the masticatory apparatus [2–4, 8, 13–15, 19, 50–53].

## Figures and Tables

**Table 1:** Differential characteristics of pain in the course of trigeminal neuralgia and temporomandibular joint dysfunction.

Common features of pain in trigeminal neuralgia and temporomandibular joint dysfunctions	Differential characteristics of pain in the course of trigeminal neuralgia and temporomandibular joint dysfunction	
	Trigeminal neuralgia	Temporomandibular joint dysfunctions
Increase in pain as a result of activity of facial or masticatory muscles	Unilateral location of pain (97%)	Bilateral pain
Possibility of pain being located on one side of the face	Characteristics of pain: acute and stabbing	Characteristics of pain: continuous and dull
Possibility of otolaryngological symptoms	Remission of the neuralgic pain during nights	Pain may still be present during the nights
Significant reduction in patients' quality of life and the development of depressive disorders	The duration of pain: very short, lasting several seconds to several minutes with long periods of remission during the day	The duration of pain: long-lasting (several hours) with short intermissions
Pain being radiated into the neighboring regions	Pain accompanied by facial muscle convulsions (tics), skin redness, lachrymation	Lack of such symptoms
